# Commentary: Facial Width-to-Height Ratio (fWHR) Is Not Associated with Adolescent Testosterone Levels

**DOI:** 10.3389/fpsyg.2016.01745

**Published:** 2016-11-18

**Authors:** Keith M. Welker, Brian M. Bird, Steven Arnocky

**Affiliations:** ^1^Department of Psychology, University of Massachusetts BostonBoston, MA, USA; ^2^Department of Psychology, Simon Fraser UniversityBurnaby, BC, Canada; ^3^Department of Psychology, Nipissing UniversityNorth Bay, ON, Canada

**Keywords:** facial structure, facial width-to-height ratio, testosterone, puberty, sex hormones, adolescence

Facial width-to-height ratio (fWHR), the ratio of the distance between the left and right zygomatic bones to the distance between the upper lip and brow, predicts many traits, displays, and behaviors, including male aggression, trustworthiness, risk-taking, and physical formidability (e.g., Carré et al., 2009; Stirrat and Perrett, 2010; Welker et al., 2015; Zilioli et al., 2015). One theorized mechanism for linking fWHR to these behavioral traits and displays is pubertal exposure to testosterone, which may reflect androgenic organizational effects on neural circuitry related to these behaviors (Carré and McCormick, 2008). Lending support to this possibility, some work suggests that low-dose administrations of testosterone modulate craniofacial growth in boys with delayed puberty (Verdonck et al., 1999), but until now, research has not examined this association.

Hodges-Simeon et al. (2016) recently examined testosterone and fWHR in 75 adolescent Tsimane males, reporting that fWHR is not associated with testosterone. However, when age was controlled for in the paper, the testosterone-fWHR association was significant and of a moderate effect size (*r*_partial_ = 0.28, *p* < 0.05). Regardless, Hodges-Simeon and colleagues conclude that these findings cast doubt on the possibility that pubertal testosterone is associated with fWHR.

We are pleased at the arrival of new research to test this predicted association. However, we note several limitations with this study. First, there is a strong association between testosterone and age reported in this paper (*r* = 0.82, *p* < 0.01). Generally, it is helpful to control for other factors that might influence an association or the involved variables (Cohen et al., 2003). With respect to testosterone and fWHR, this could include age, body-mass index, genetic influences, and physical health and exercise (e.g., Zitzmann and Nieschlag, 2001; Hehman et al., 2014; Geniole et al., 2015; Shaffer et al., 2016). Therefore, the authors' analysis should include an age covariate, since it is critical to account for strong developmental effects on the variables of interest. Furthermore, it is important to transform highly skewed variables such as testosterone when performing conventional statistical analyses such as correlation. Although Hodges-Simeon and colleagues perform these transformations in later regression analyses, these transformations were not used for correlations. Finally, Hodges-Simeon and colleagues also used a liberal criterion for adolescence in their sample (ages 8–22) to assess whether fWHR is associated with pubertal testosterone. The pubertal growth spurt and sexual maturation for boys typically occurs between the ages of 12–16 years (Tanner and Whitehouse, 1976), suggesting that the authors' decision to precede the earlier age by 4 years and exceed the later age by 6 years is representative of childhood to adulthood, rather than adolescence or puberty.

Using the supplemental data provided, we re-examined the correlation between fWHR and testosterone, controlling for age. Following the paper's exclusion criteria, outliers (*N* = 4) and cases with missing saliva samples (*N* = 6) or facial pictures (*N* = 6) were identified and removed^1^. Additionally, testosterone and age were log-transformed to correct skewness. Across all data, fWHR and testosterone were significantly correlated when controlling for age [*r*_partial_(70) = 0.25, *p* = 0.035], as similarly reported by Hodges-Simeon and colleagues. When restricting this correlation to those within the 12–16 age range, the association was also significantly positive [*r*_partial_(30) = 0.35, *p* = 0.049]. To illustrate this, Figure 1 presents a scatterplot of the association between fWHR and testosterone, adjusted for age. Some research suggests that the typical adolescent growth-spurt in the Tsimane may occur between the ages of 12 and 18 years (Walker et al., 2006). Within this age range, we found that the testosterone-fWHR association (still controlling for age) was of similar effect size to the analysis with the full data, but non-significant [*r*_partial_(37) = 0.24, *p* = 0.141]. Although the non-significance of this effect is likely due to diminished statistical power, it is nonetheless compelling that the magnitude of the effect size remains similar to that from the full dataset. Additionally, we also regressed log-transformed testosterone on each of the measured facial characteristics (fWHR, facial width to lower face height ratio, lower face to full face ratio, and log-transformed cheekbone prominence) in the full “peri-pubertal” sample, controlling for log-transformed age. In this model (*R*^*2*^_adjusted_ = 0.72), fWHR was positively associated with testosterone (β = 0.32, *t*_(67)_ = 3.75, *p* < 0.001), uniquely accounting for 17% of testosterone's variability ($r_{\text{partial}}^{2}=$ 0.17). Facial width to lower face height ratio negatively predicted testosterone (β = −0.38, *p* = 0.004), whereas age and cheekbone prominence positively predicted testosterone (β = 0.69, *p* < 0.001 and β = 0.17, *p* = 0.049, respectively). In summary, when the proper covariates are included and the assumptions of the analyses are accounted for, there is a positive association between fWHR and pubertal testosterone.

**Figure 1:**
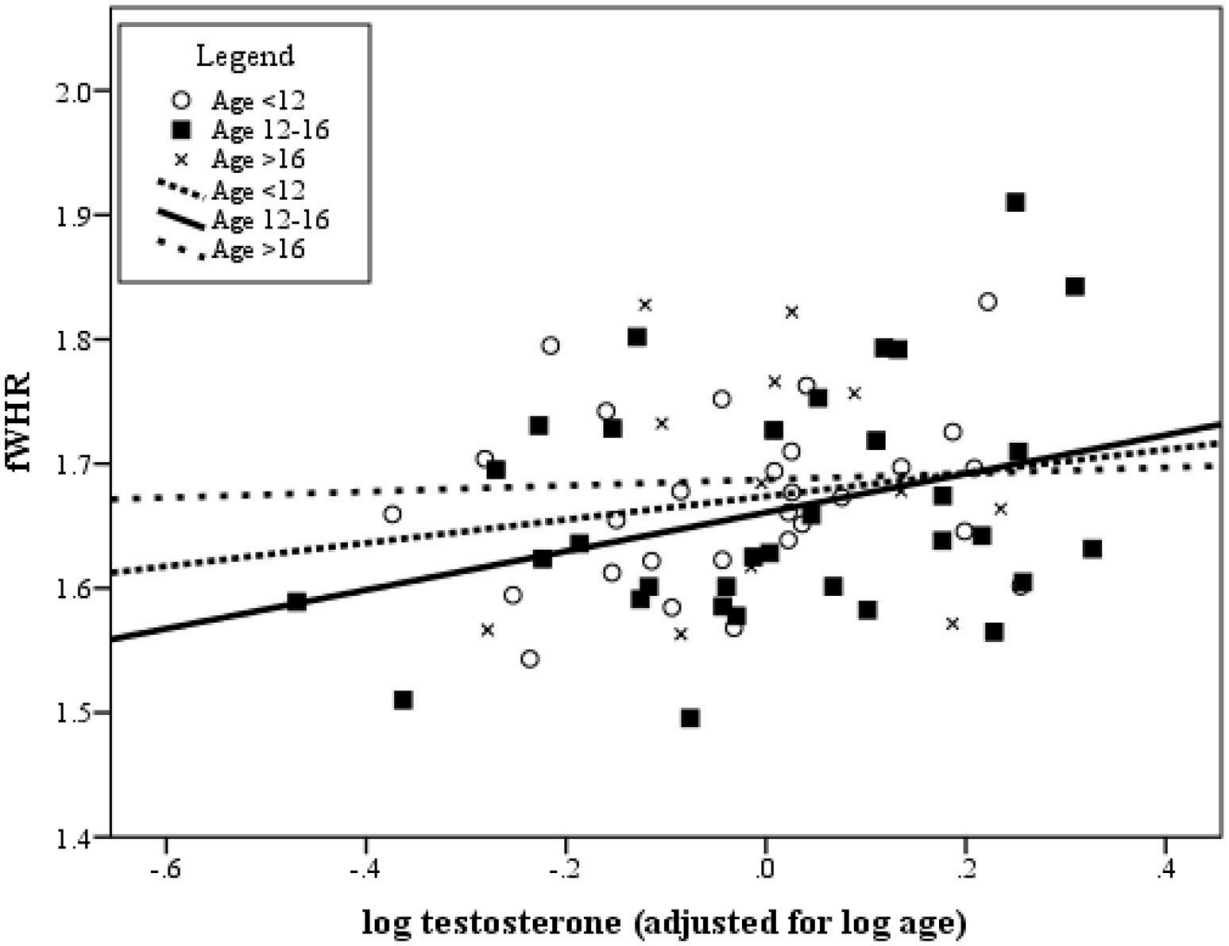
**Scatterplot of the association between fWHR and adolescent testosterone (adjusted for age)**. Data are split into age ranges (<12 years, between 12–16 years, and 16+ years). Testosterone values were adjusted for age by regressing log-transformed testosterone on log-transformed age and saving the unstandardized residuals.

Altogether, the examination of associations between pubertal testosterone and facial structure needs more evaluation from further studies. In particular, larger sample sizes are needed to estimate the association between pubertal testosterone and fWHR with greater confidence. Future research also needs to account for critical covariates, assumptions of statistical analyses, and age ranges of puberty. Although, based on these initial data, it appears that pubertal testosterone explains between about 6 to 17% of the variability in fWHR, there are likely influences and correlates other than testosterone predicting facial structure that need to be accounted for (e.g., genetic factors, physical exercise, body mass). Additionally, given the emerging findings of fWHR predicting social behavior and perceptions and the limitations of cross-sectional designs, future longitudinal research is needed to examine how pubertal testosterone and cranial growth predict social behaviors in adulthood. This longitudinal research will be able to provide a more powerful test of the relationship between intra-individual changes in pubertal testosterone and fWHR.

## Author contributions

KW wrote the commentary and performed data analysis. BB provided edits and suggestions for revision. SA performed data analysis and provided edits and suggestions.

### Conflict of interest statement

The authors declare that the research was conducted in the absence of any commercial or financial relationships that could be construed as a potential conflict of interest.

## Footnotes

^1^Although reasons for exclusion were not included in the supplemental data, we inferred that the six data points with substantial missing data and no fWHR data were those with missing photographs. We did note, however, two additional data points missing fWHR. Altogether, analysis with fWHR did not include these eight data points.
